# Unfractionated and Low-Molecular-Weight Heparin and the Phosphodiesterase Inhibitors, IBMX and Cilostazol, Block *Ex Vivo* Equid Herpesvirus Type-1-Induced Platelet Activation

**DOI:** 10.3389/fvets.2016.00099

**Published:** 2016-11-17

**Authors:** Tracy Stokol, Priscila B. S. Serpa, Muhammad N. Zahid, Marjory B. Brooks

**Affiliations:** ^1^Department of Population Medicine and Diagnostic Sciences, Cornell University, Ithaca, NY, USA

**Keywords:** equine, EHV-1, thrombin generation, thrombosis, P-selectin, flow cytometry, horse

## Abstract

Equid herpes virus type-1 (EHV-1) is a major pathogen of horses, causing abortion storms and outbreaks of herpes virus myeloencephalopathy. These clinical syndromes are partly attributed to ischemic injury from thrombosis in placental and spinal vessels. The mechanism of thrombosis in affected horses is unknown. We have previously shown that EHV-1 activates platelets through virus-associated tissue factor-initiated thrombin generation. Activated platelets participate in thrombus formation by providing a surface to localize coagulation factor complexes that amplify and propagate thrombin generation. We hypothesized that coagulation inhibitors that suppress thrombin generation (heparins) or platelet inhibitors that impede post-receptor thrombin signaling [phosphodiesterase (PDE) antagonists] would inhibit EHV-1-induced platelet activation *ex vivo*. We exposed platelet-rich plasma (PRP) collected from healthy horses to the RacL11 abortigenic and Ab4 neuropathogenic strains of EHV-1 at 1 plaque-forming unit/cell in the presence or absence of unfractionated heparin (UFH), low-molecular-weight heparin (LMWH) or the PDE inhibitors, 3-isobutyl-1methylxanthine (IBMX), and cilostazol. We assessed platelet activation status in flow cytometric assays by measuring P-selectin expression. We found that all of the inhibitors blocked EHV-1- and thrombin-induced platelet activation in a dose-dependent manner. Platelet activation in PRP was maximally inhibited at concentrations of 0.05 U/mL UFH and 2.5 μg/mL LMWH. These concentrations represented 0.1–0.2 U/mL anti-factor Xa activity measured in chromogenic assays. Both IBMX and cilostazol showed maximal inhibition of platelet activation at the highest tested concentration of 50 μM, but inhibition was lower than that seen with UFH and LMWH. Our results indicate that heparin anticoagulants and strong non-selective (IBMX) or isoenzyme-3 selective (cilostazol) PDE antagonists inhibit *ex vivo* EHV-1-induced platelet activation. These drugs have potential as adjunctive therapy to reduce the serious complications associated with EHV-1-induced thrombosis. Treatment trials are warranted to determine whether these drugs yield clinical benefit when administered to horses infected with EHV-1.

## Introduction

Equid herpesvirus type-1 (EHV-1) is a major contagious pathogen of horses, causing individual cases and outbreaks of respiratory disease worldwide. The most severe clinical consequences of EHV-1 infection are abortion and a neurologic syndrome, called equine herpes myeloencephalopathy (EHM). Horses suffering from EHM are frequently euthanized due to the severity of neurologic symptoms and performance horses affected with milder neurologic disease often have reduced performance (1). Abortion and EHM are thought to result from EHV-1 infection of placental and neural tissue, with the virus reaching these tissues through a cell-associated viremia and subsequent endothelial infection, vasculitis, and tissue invasion (2–5). Pathologic studies in experimentally and naturally infected horses have revealed the presence of thrombi in placental and neural vessels (2, 5, 6). Hypoxic damage from thrombosis likely contributes substantially to the tissue injury that results in abortion and EHM.

Horses experimentally infected with EHV-1 have high d-dimer concentrations during the viremic phase of infection (7). d-dimer is the terminal breakdown product of cross-linked fibrin and high concentrations support a systemic hypercoagulable and prothrombotic state (8). The cause of the documented hypercoagulability and thrombosis in EHV-1-infected horses is unknown, but is likely multifactorial, involving release or upregulation of procoagulants, such as tissue factor, with endothelial injury or vasculitis. We have also previously shown that EHV-1 induces tissue factor expression in equine monocytes (9). Because tissue factor is the main activator of coagulation *in vivo* (10), upregulated expression of tissue factor on monocytes could be one trigger of the hypercoagulable state. We have also recently shown that EHV-1 alone can activate coagulation and generate thrombin in equine plasma *ex vivo* (11). Thrombin generation was initiated by tissue factor expressed on the virus, with the tissue factor presumably being incorporated into the virus envelope during budding from the propagating cell line. We also found that the virus-generated thrombin activated platelets in equine platelet-rich plasma (PRP), causing α-granule exteriorization, characterized by surface expression of the α-granule protein, P-selectin, and release of membrane-derived microparticles (11).

Platelets play an important role in thrombosis. Once activated, they not only form dense fibrinogen-bound aggregates but also mobilize lipid membranes, providing a phosphatidylserine-rich outer membrane surface that catalyzes thrombin generation (so-called platelet procoagulant activity) (12). In accordance with this, we have found that addition of platelets to equine platelet-poor plasma (PPP)-containing EHV-1 generated more thrombin than the virus in PPP alone (11). Activated platelets also help recruit and bind leukocytes to the developing thrombus by forming adhesive bonds between platelet surface-expressed P-selectin and leukocyte-expressed P-selectin glycoprotein ligand-1 (13). Once bound, leukocytes promote thrombus formation by expressing tissue factor (monocytes) or undergoing NETosis (neutrophil extracellular traps) (10, 14). Thus, inhibiting platelet activation and particularly P-selectin expression could substantially reduce thrombus formation and may provide therapeutic or prophylactic options for horses at-risk of abortion and EHM due to EHV-1 infection.

We recently performed a clinical trial in horses to determine whether traditional antiplatelet drugs, including aspirin and the ADP receptor antagonist, clopidogrel, could inhibit EHV-1-induced platelet activation. We also tested the non-specific phosphodiesterase (PDE) inhibitors, theophylline and pentoxifylline, which are weak blockers of platelet signaling downstream of receptor activation (15). We found that none of these drugs, when given to horses at standard therapeutic doses, were effective against EHV-1-induced platelet activation *ex vivo*.^1^ This finding can be attributed to the inability of these antiplatelet drugs to prevent thrombin generation, which is the primary mechanism underlying the EHV-1-induced activation response. Our results indicate a need to find inhibitors that effectively block the thrombin-mediated platelet-activating effects of EHV-1.

We hypothesized that inhibition of thrombin generation or stronger and more selective inhibition of thrombin-mediated platelet signaling could block EHV-induced platelet activation. To test this hypothesis, we added anticoagulant and antiplatelet agents to PRP obtained from healthy horses and then measured platelet activation after *ex vivo* exposure to EHV-1. Flow cytometric detection of α-granule release based on surface P-selectin expression was used as a marker of platelet activation. To inhibit thrombin generation, we tested unfractionated heparin (UFH) and low-molecular-weight heparin (LMWH), anticoagulants that are used clinically for thromboprophylaxis in horses (16, 17). To inhibit thrombin-induced platelet signaling, we tested the strong competitive non-selective PDE inhibitor, 3-isobutyl-1methylxanthine (IBMX) (18), and the selective PDE isoenzyme 3 (PDE3) inhibitor, cilostazol (15). High concentrations of intraplatelet cAMP and cGMP act as a brake against agonist-initiated signaling that culminates in platelet activation. Phosphodiesterases normally direct the hydrolysis of cAMP, maintaining low intracellular cAMP and cGMP concentrations, which then permits signaling induced by various agonists, including thrombin, adenosine diphosphate, and platelet-activating factor. PDE inhibitors increase intracellular cAMP and cGMP concentrations and block platelet activation downstream of agonist receptor engagement (15, 18–21). Several isoenzymes of PDE have been identified in horses, of which PDE3 has been ascribed the main role in blocking platelet activation secondary to agonists (18). We chose IBMX and cilostazol because they effectively inhibit P-selectin expression and platelet aggregation in agonist-stimulated equine (IBMX) (18, 19) and human platelets (22) *ex vivo*, respectively.

## Materials and Methods

### Virus Isolation

The RacL11 [originally isolated from an aborted fetus (23)] and Ab4 [originally isolated from a gelding with equine herpesvirus myeloencephalopathy (6)] strains of EHV-1 were purified from rabbit kidney 13 cell lysates using sucrose gradient ultracentrifugation, as previously described (11). Purified virus was reconstituted in phosphate-buffered saline (PBS) and frozen at −80°C in aliquots with single use thaws. Virus stocks were titered using a standard plaque assay with rabbit kidney 13 cells.

### Blood Sample Collection and Preparation of Platelet-Rich and Platelet-Poor Plasma

Both PRP and PPP were prepared from 4.5 mL of blood collected directly into a 6-mL syringe containing 0.5 mL 3.8% citrate from the jugular vein of clinically healthy horses housed at the Cornell University Equine Research Park as previously described (11). The horses consisted of 16 mares and 2 stallions. None of the mares were pregnant or in the first month of lactation. In brief, PRP was prepared within 15–30 min of collection by low-speed centrifugation (250 × *g*, 21°C, 10 min) of the leukocyte-PRP resulting from gravity sedimentation of erythrocytes for 20 min at 20–23°C (room temperature). A platelet count was performed on the resulting PRP with an automated hematology analyzer (ADVIA 2120, Siemens Healthcare Diagnostics Inc., Tarrytown, NJ, USA). Platelet-poor plasma was prepared by high-speed centrifugation of residual PRP (15,000 *g*, 20–23°C, 5 min). The sample collection protocol was approved by the Institutional Animal Care and Use Committee at Cornell University (#2007-0086), which follows several federal and state guidelines.

### Platelet Exposure to Virus and Inhibitors

The PRP was diluted (1 × 10^6^/mL, final concentration) in flow buffer (10 mM HEPES, 140 mM NaCl, 2.5 mM calcium chloride, pH 7.4, with 20 mM supplemental glycine–proline–arginine–proline to inhibit fibrin polymerization) to a final volume of 100 μL. Platelets were exposed to each virus strain at 1 plaque-forming unit (PFU)/cell for 10 min at 37°C, with PBS and bovine thrombin (0.15 U/mL; 20–30 nM, Sigma-Aldrich, St. Louis, MO, USA) as negative and positive controls of platelet activation, respectively. Inhibitors were added to the buffer before addition of PRP. The following inhibitors were used: UFH (Sagent Pharmaceuticals, Schaumburg, IL, USA) at final concentrations ranging from 0.02 to 2 U/mL; LMWH (powder, Galen Laboratory Supplies, Middletown, CT, USA) at final concentrations ranging from 0.1 to 5.0 μg/mL; IBMX (Sigma-Aldrich) at final concentrations ranging from 5 to 50 μM; and cilostazol (Sigma-Aldrich) at final concentrations ranging from 1 to 50 μM. For UFH and LMWH, PBS was the vehicle control, whereas for the PDE inhibitors, dimethylsulfoxide (DMSO) was the vehicle control. The PRP from individual horses (or experiments) was not spiked with all drug concentrations, however, negative and positive controls were included in each experiment and a minimum of three replicates (three different horses) of each concentration was tested. In two horses, the thrombin positive control did not work due to a dilutional error or the inhibitor was mistakenly not added to the PBS control. The data for these controls were not included in the provided results. Chondroitin sulfate (5 μg/mL, type A sodium salt from bovine trachea, Sigma-Aldrich) is a negatively charged polysaccharide and was used as a negative control for UFH and LMWH. For LMWH, the higher tested concentrations (2.5–5.0 μg/mL) were equivalent to the total maximal reported plasma concentrations (0.24–0.45 U/mL) obtained with pharmacokinetic studies in horses using doses of 40–100 U/mL (24). For cilostazol, concentrations were based on that previously used to inhibit P-selectin expression in human platelets after thrombin or collagen stimulation (22). For IBMX, concentrations were selected based on previous studies with human (21) and equine (18, 19) platelets. We chose cilostazol over trequinsin, a PDE3 inhibitor that has been used in previous studies in horses (18–20), due to cost.

### Flow Cytometric Detection of Platelet Activation

**Figure 1:**
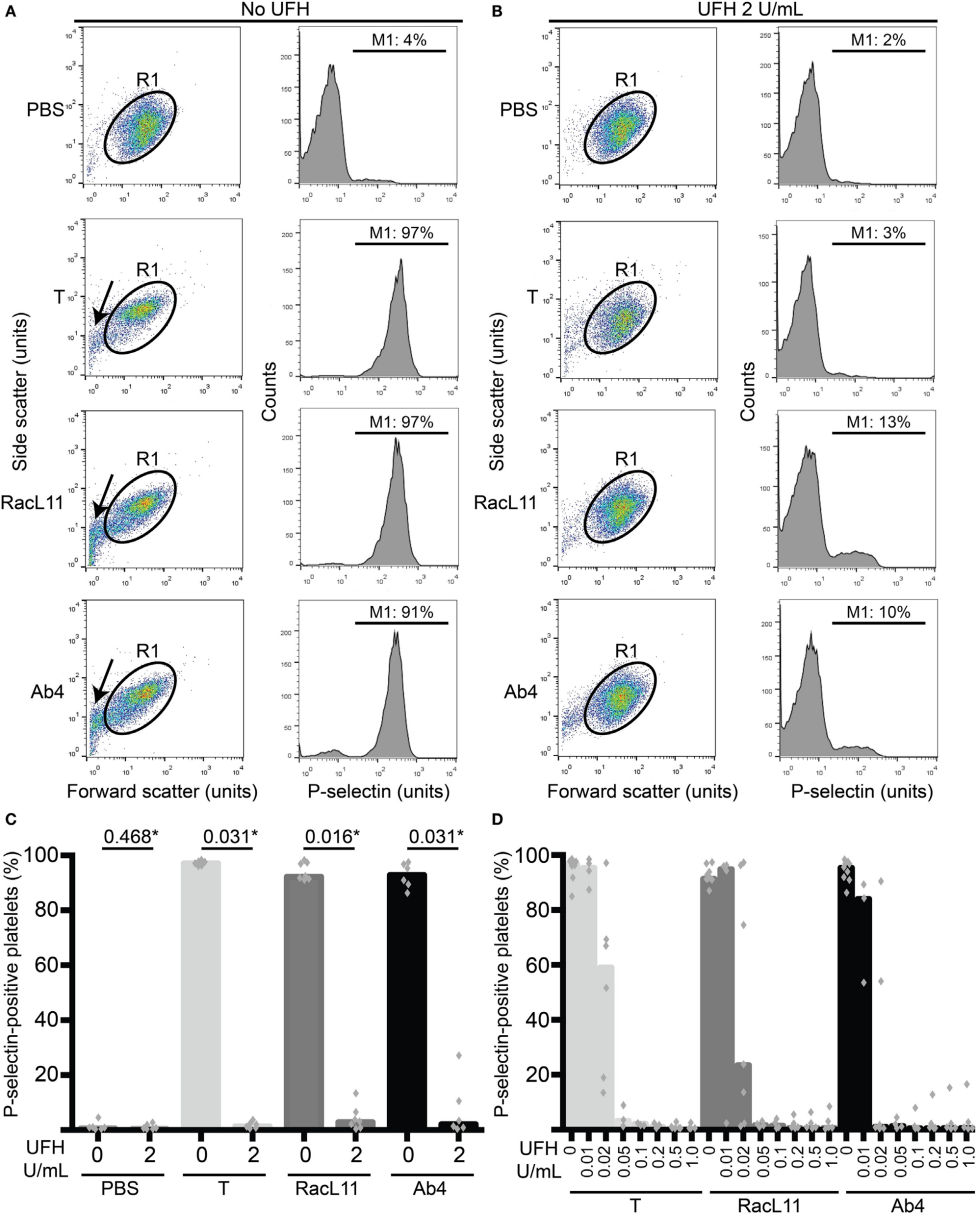
**Unfractionated heparin (UFH) inhibits EHV-1-induced platelet activation, as measured by P-selectin expression with flow cytometry**. (A) Platelets were gated (R1) in a forward versus side scatter plot (left panel) and the percentage of P-selectin-positive cells in the gated region was measured (marker or M1 region) using histogram plots of P-selectin fluorescence (right panel). In the absence of UFH, thrombin (0.15 U/mL) and both RacL11 and Ab4 strains of EHV-1 at 1 PFU/cell activated platelets. Activation was evident as lengthening and narrowing of the platelet event cloud, with some microvesiculation (arrows) and >90% platelets expressing P-selectin. **(B)** In the presence of 2 U/mL UFH, inhibition of platelet activation is seen in forward versus side scatter (left panel) and histogram plots of P-selectin expression (right panel) in gated platelets (R1) exposed to thrombin and both viruses. No changes are seen in the PBS control with or without UFH. Residual small events (<10^1^ forward scatter units) in the virus-exposed samples in the presence of UFH likely represent aggregated virus particles. Images in **(A,B)** are representative of one horse. Note that EHV-1-induced activation in this horse was not completely abolished in the presence of heparin, possibly due to mild “preactivation” of platelets (4% P-selectin expression in PBS control). **(C)** Quantification of the median percentage of P-selectin-positive platelets (columns), with superimposed individual data points, in response to thrombin (light gray columns) or both strains of EHV-1 (RacL11, dark gray columns; Ab4, black columns) in the absence or presence of 2 U/mL UFH (*n* = 6–7). *Numbers shown are *P* values (Wilcoxon matched pairs sign rank). **(D)** A heparin dose titration curve showed consistent inhibition of thrombin (light gray columns) and EHV-1-induced platelet activation at 0.05 U/mL (RacL11, dark gray columns; Ab4, black columns; *n* = 3–10). Columns represent medians with superimposed individual data points.

Flow cytometric measurement of P-selectin on platelet surfaces, indicating degranulation of α-granules, was used as an indicator of platelet activation, as we have previously described (11). In brief, after 10 min of exposure to virus or activation controls, platelets were incubated with an antibody against P-selectin conjugated to Alexa-647 or allophycocyanin (33.3 ng/mL final concentration, clone Psel.KO.2.7, Novus Biologicals, Littleton, CO, USA) for 10 min, followed by quench dilution (400 μL) in flow buffer. The samples were then analyzed with a flow cytometer (FACSCalibur™, BD Biosciences, Franklin Lakes, NJ, USA), gating platelets on their characteristic forward and side scatter. The percentage of P-selectin-positive platelets was then determined from histogram plots of the gated platelets (Figure 1A), using commercial software (FlowJo v10, FlowJo LLC, Ashland, OR, USA).

### Measurement of Anti-Factor Xa Activity

To relate the concentrations of UFH and LMWH added to PRP to target anticoagulant effects of heparin measured by factor Xa inhibitory activity (anti-Xa), PPP samples collected from the study horses were spiked with the drugs and frozen at −20°C until batch assay. Anti-Xa activities were measured using a commercial kit (Liquid anti-Xa, Diagnostica Stago) and an automated coagulation analyzer (STA Compact, Diagnostica Stago, Parsippany, NJ, USA). The assay is a one-step competitive inhibition assay, configured with a bovine factor Xa reagent and a chromogenic substrate of factor Xa. The color change of the reaction mixture is inversely proportional to the heparin concentration in the test plasma (expressed as units per milliliter anti-Xa). The assay calibrators contain known concentrations of UFH or LMWH in human plasma (STA-calibrator HBPM/LMWH, Diagnostica Stago). Routinely used target anti-Xa activities in horses (based on heparin therapy in humans) are 0.1–0.2 U/mL anti-Xa for prophylaxis and 0.3–0.7 U/mL anti-Xa for therapy (24).

### Statistical Analysis

Data were non-parametric and results are expressed as median, with range as applicable. Graphical displays are column charts with medians and superimposed individual data points. For many experiments, obtained results were consistent between horses but could not be statistically compared due to the number of repeats (i.e., less than six different horses per experiment). Thus only selected results (those with sufficient number of replicates) were compared with a Wilcoxon match paired sign rank test. Alpha was set at 5% (two-tailed).

## Results

We first determined whether UFH could inhibit EHV-1-induced platelet activation. We found that UFH at a concentration of 2 U/mL spiked into PRP inhibited platelet activation induced by both strains of EHV-1 at 1 PFU/cell and by the positive control treatment of 0.15 U/mL of thrombin. This inhibition was evident by minimal changes in the platelet event cloud on flow cytometric forward versus side scatter plots and a marked significant reduction in platelet P-selectin expression on histogram plots (Figures 1A–C). When spiked into equine PPP, 2 U/mL UFH yielded anti-Xa activities above target range therapeutic anti-Xa activity (0.3–0.7 anti-Xa U/mL) (Table 1). To relate the UFH concentrations that inhibited EHV-1-induced platelet activation with target range anticoagulant anti-Xa activities, we performed a dose-response series. We found that EHV-1-induced platelet activation was inhibited by UFH in PRP of all tested horses at a concentration of 0.05 U/mL, with variable inhibition among individuals at UFH concentrations of 0.02 U/mL. Some differences were noted between virus strains at the lowest UFH concentrations (Figure 1D). A concentration of 0.1 U/mL UFH was required to consistently inhibit thrombin-induced platelet activation, with PRP from some horses showing partial inhibition at 0.05 U/mL UFH (Figure 1D). Anti-Xa activity was detectable in the PPP of two out of three horses spiked with 0.1 U/mL UFH and was generally within target range of 0.3–0.7 anti-Xa U/mL at UFH concentrations ranging from 0.2 to 1 U/mL UFH (Table 1). The EHV-1-stimulated PRP of one horse demonstrated an “escape” from inhibition with up to 8 and 16% P selectin expression persisting in RacL11- and Ab4-activated reactions containing increasing concentrations of UFH (Figure 1D). A similar “escape” was not seen after thrombin stimulation in this horse, arguing against a dilution error. The reason for this finding is unclear (the other inhibitors were not tested in this horse).

**Table 1:** **Median and range of anti-factor Xa activity in equine platelet-poor plasma spiked with various doses of unfractionated heparin (UFH)**.

UFH dose (U/mL)	Anti-Xa activity (U/mL)		Number of horses
	Median	Range	
0	0	0–0	9
0.02	0	0–0	6
0.05	0	0–0	4
0.1	0	0–0.1	3
0.2	0.1	0.1–0.2	5
0.5	0.3	0.2–0.6	4
1	0.7	0.6–0.8	4
2	1.5	1.4–2.9	5

**Figure 2:**
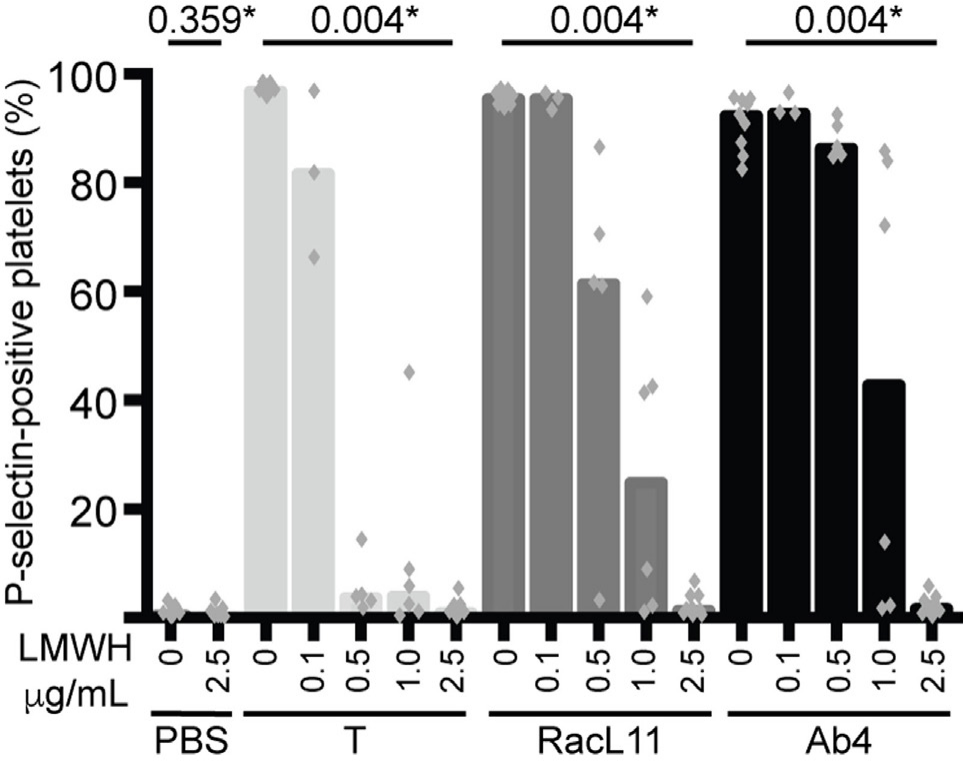
**Low-molecular-weight heparin (LMWH) inhibits EHV-1-induced platelet activation, as measured by P-selectin expression with flow cytometry**. P-selectin expression was quantified in response to thrombin (0.15 U/mL, light gray columns) or RacL11 (dark gray columns) and Ab4 (black columns) strains of EHV-1 at 1 PFU/cell as the percentage of gated platelets in the absence or presence of increasing concentrations of LMWH (0.1–2.5 μg/mL) (*n* = 3–9). Complete inhibition was observed at 2.5 μg/mL (*P values compared to no LMWH, Wilcoxon matched pairs sign rank) and 5 μg/mL (not shown). No activation was seen with the PBS control in the absence or presence of LMWH (only highest dose shown). Columns represent medians with superimposed individual data points.

We next tested whether various concentrations of LMWH could inhibit EHV-1-induced platelet activation. We found that inhibition of EHV-1-induced platelet activation began at 0.5 μg/mL and was complete at 2.5 μg/mL LMWH for both strains. Virus strain-dependent differences were seen in the inhibitory activity of LMWH, with RacL11 showing a trend toward enhanced susceptibility to low-dose LMWH inhibition than Ab4 (Figure 2). Target prophylactic anti-Xa activities were only achieved in all horses at 2.5 μg/mL LMWH (Table 2), with only one out of three tested horses achieving a target therapeutic anti-Xa activity at this concentration. At the highest tested dose of 5 μg/mL, which completely blocked EHV-1- and thrombin-induced activation (not shown), target therapeutic concentrations were obtained in two out of three tested horses (Table 1). In contrast to UFH, LMWH showed a trend toward stronger inhibition of thrombin-induced activation compared to the activation caused by both virus strains, with inhibition of thrombin activation starting at 0.1 μg/mL LMWH and being almost complete at 0.5 μg/mL (Figure 2). Exposure of PRP to chondroitin sulfate, a glycosaminoglycan with no anticoagulant action used as a negative control for heparin, had no effect on thrombin- or EHV-1-induced platelet activation (Figure S1 in Supplementary Material).

**Table 2:** **Median and range of anti-factor Xa activity in equine platelet-poor plasma spiked with various doses of low-molecular-weight heparin (LMWH)**.

LMWH dose (μg/mL)	Anti-Xa activity (U/mL)		Number of horses
	Median	Range	
0	0	0–0	5
0.1	0	0–0	2
0.5	0	0–0	3
1	0	0–0.1	3
2.5	0.1	0.1–0.6	4
5.0	0.3	0.2–0.3	3

**Figure 3:**
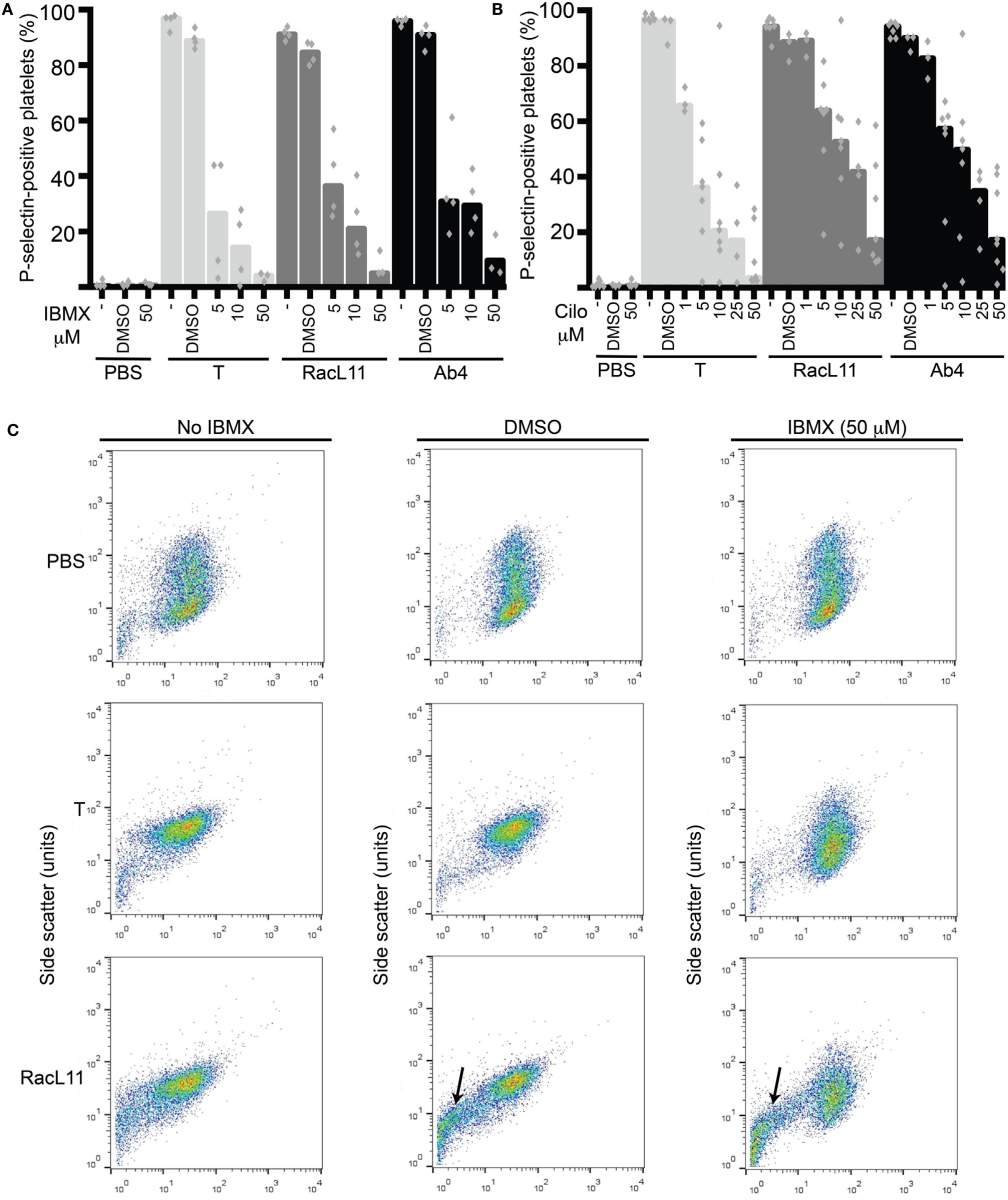

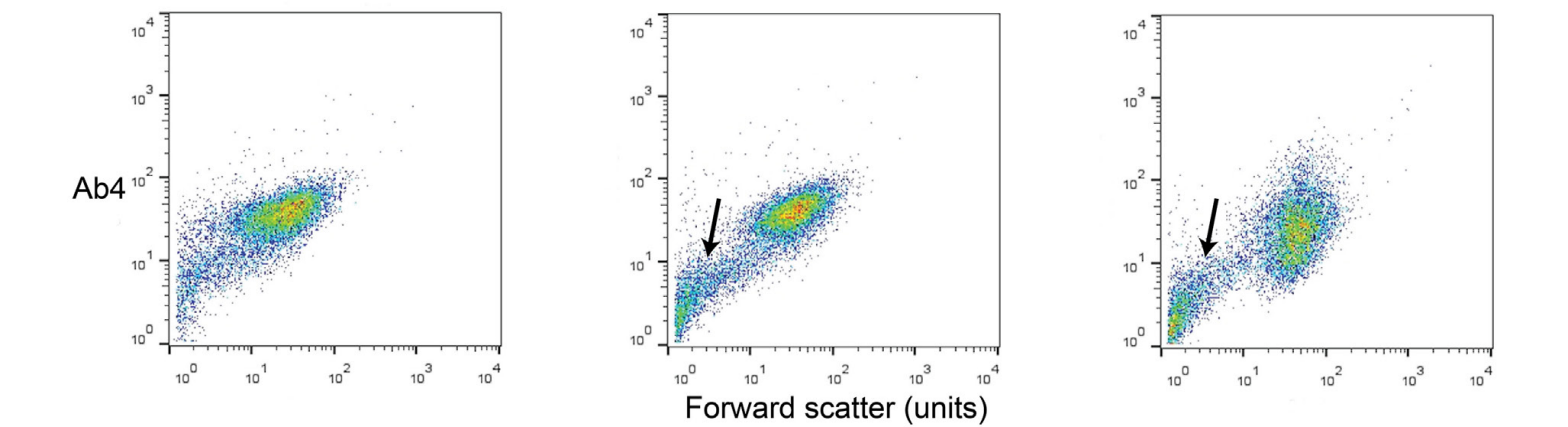
**The phosphodiesterase inhibitors, IBMX and cilostazol, reduce EHV-1-induced platelet activation, as measured by P-selectin expression with flow cytometry**. P-selectin expression in response to thrombin (0.15 U/mL, light gray columns) or RacL11 (dark gray columns) and Ab4 (black columns) strains of EHV-1 at 1 PFU/cell was quantified as the percentage of gated platelets in the absence or presence of increasing concentrations of **(A)** IBMX (5–50 μM) (*n* = 3–4) or **(B)** cilostazol (cilo, 1–50 μM) (*n* = 3–9). Both drugs inhibited activation induced by thrombin and both virus strains, to varying degrees. The DMSO vehicle control mildly suppressed the activation responses. No activation was seen with the PBS control in the absence or presence of either drug (only highest dose of drug shown). Bars represent medians with superimposed individual data points. **(C)** Forward versus side scatter plots show the inhibitory effects of IBMX as well as the platelet fragmentation or vesiculation that occurred in the presence of the DMSO vehicle and virus (arrows), but not thrombin. A similar vesiculation effect was seen with cilostazol (not shown). Increased small events (<10^1^ forward scatter units) in the virus alone-exposed samples represent a combination of microvesicles or platelet fragments and aggregates of virus particles. The number of replicates was insufficient to perform statistical analysis.

We then tested the strong non-selective PDE inhibitor, IBMX, and the selective PDE3 inhibitor, cilostazol, for their inhibitory effect on EHV-1-induced platelet activation. We found that both inhibitors reduced platelet activation induced by EHV-1 and our thrombin positive control, with IBMX showing a trend toward a stronger effect than cilostazol at similar concentrations. There was a trend for thrombin stimulation to be inhibited by a greater degree than virus exposure at the same concentration of PDE inhibitors (Figures 3A,B). The presence of DMSO vehicle or PDE inhibitors did not enhance platelet activation in negative (PBS) control reactions. We found that the DMSO vehicle displayed a mild, but not significant, suppressive effect on activation induced by EHV-1 and thrombin (Figures 3A,B). We also noticed that the DMSO vehicle and both PDE inhibitors caused microvesiculation or fragmentation of platelets in the presence of EHV-1, but not thrombin (Figure 3C).

## Discussion

In this *ex vivo* study, we found that anticoagulants that inhibit thrombin generation (UFH, LMWH) and antiplatelet drugs that inhibit signaling pathways downstream of agonist receptors (IBMX, cilostazol) block EHV-1-induced platelet activation, as measured by platelet P-selectin expression. Because ischemic injury from thrombosis contributes to the EHV-1-associated clinical syndromes of abortion and EHM and platelets are crucial for thrombus formation, our results suggest that these drugs may be useful to prevent or ameliorate EHV-1-induced thrombosis in at-risk horses. Notably, EHV-1-induced platelet activation at 1 PFU/cell was inhibited by UFH doses that did not yield detectable anti-Xa activity when spiked into equine PPP. Similar results were seen with LMWH, although complete inhibition of EHV-1-induced activation required LMWH doses that yielded higher anti-Xa activities (0.1–0.2 U/mL). These data suggest that low doses of both types of heparin may be sufficient to inhibit EHV-1-induced platelet activation *in vivo*. However, the number of virions that platelets may be exposed to *in vivo* is unknown. It is possible that at sites of high virus replication, such as infected endothelial cells, platelets may be exposed to more than 1 PFU/cell and higher circulating levels of heparin anti-Xa activity may be required.

We observed a trend for UFH to have a stronger inhibitory effect on EHV-1- than thrombin-induced platelet activation. This differential inhibitory effect was most apparent at the lower UFH concentrations of 0.02 and 0.05 U/mL. A potential explanation for this observation is that heparin may inhibit EHV-1 binding to platelets, as reported for Dengue virus (25). Since the envelope glycoproteins gB and gC of equine α-herpesviruses, including EHV-1 and EHV-4, can bind to heparin-like glycosaminoglans on cells, heparin can be used to block virus binding to cells (26–28). We have previously found that EHV-1 does associate with platelets (11) and speculate that this association is mediated through the virus tethering to heparin-like glycosaminoglycans *via* gB and gC, although other virus-platelet receptor interactions may be involved. Binding of tissue factor-expressing virions to a phosphatidylserine-rich cell surface expressed on activated platelets would substantially boost thrombin generation, as we have shown when we added virions to equine PRP in a thrombin generation assay (11). Based on these previous observations, we speculate that UFH may be inhibiting virus attachment to platelet surfaces, limiting thrombin generation to “free” or unattached virions, which would then generate less thrombin overall. This effect may only become evident at lower UFH concentrations, which are insufficient to completely block any free thrombin generated by the virus. An alternative explanation is that EHV-1, at 1 PFU/cell, is generating less thrombin in equine PRP than 0.15 U/mL of exogenous thrombin. However, this explanation is not supported by our results with LMWH, which showed a stronger inhibitory effect for thrombin- than EHV-1-induced platelet activation with LMWH at doses less than 2.5 μg/mL. A similar trend was seen with the PDE inhibitors, IBMX, and cilostazol. The latter data suggest that EHV-1 is generating more thrombin than the exogenously added thrombin in our positive control, which is then insufficiently inhibited by lower LMWH or PDE inhibitor concentrations. It is also possible that LMWH, being of shorter length and less charge, is a less effective inhibitor of virus binding to heparin-like glycosaminoglycans than UFH. Chondroitin sulfate, which is negatively charged like heparin and is used as a negative control for heparin in virus-cell binding studies (25), had no effect on EHV-1-induced platelet activation.

We observed some differences between the 2 tested virus strains in the degree of inhibition in response to the various drugs in the same horse. For LMWH at 0.5–1 μg/mL, there was a trend toward a larger degree of inhibition for RacL11- than Ab4-induced activation. This observation is consistent with our previous finding that Ab4 generates more thrombin *ex vivo* at the same PFU/cell than RacL11 (11). However, the opposite finding was true for the lower concentrations of UFH and cilostazol, whereas a clear pattern was not apparent with IBMX, the strong PDE inhibitor. The reason for these within horse differences in the inhibitory effect of the drugs against platelet activation induced by the two virus strains is unclear. Since heparin may inhibit virus binding as well as thrombin generation and cilostazol is a post-signaling antagonist, it is possible that there are virus strain-dependent differences in binding to platelets, as we have speculated previously (11).

Substantial variability was also evident between individual horses in the degree of suppression of EHV-1-induced platelet activation with all inhibitors. This can likely be explained by individual differences in coagulation factor concentrations, which are required by the virus to generate thrombin. A similar biological variability in the suppression of thrombin generation by various drugs, including heparin, has been reported in healthy human volunteers (29). This variability can be partly explained by documented differences between healthy individuals in thrombin generation (30), potentially due to variable coagulation factor concentrations and platelet procoagulant activity (31). We have seen similar variability in thrombin generation in horses (unpublished observations). Inter-horse variability was also observed in the suppressive effect of the drugs on thrombin-induced platelet activation. This could potentially be explained by the exogenous thrombin triggering amplification of the horse’s own coagulation factors in the recalcified citrated plasma. However platelet-dependent differences in response to agonists, such as differential calcium signaling or other genetic or environmental factors (32, 33), may also explain the observed variability between horses in the response to the inhibitors.

The PDE inhibitors, IBMX and cilostazol, inhibited EHV-1- and thrombin-induced platelet activation in equine PRP. At equivalent concentrations, IBMX generally had a stronger inhibitory effect than cilostazol on EHV-1 and thrombin-induced platelet activation. This is likely because IBMX is a strong non-selective PDE inhibitor (18), whereas cilostazol is a selective PDE3 inhibitor. Even though PDE3A appears to be the main isoform responsible for thrombin-induced platelet activation (21), other PDE isoforms or isoenzymes likely contribute to activation (15). Cilastazol showed a similar partial inhibition of thrombin-stimulated P-selectin expression in human platelets at comparable concentrations to that used herein (22). Trequinsin, another PDE3 inhibitor, also inhibits thrombin-induced activation of equine platelets (18–20), however, to our knowledge, this is the first study testing cilostazol for platelet inhibitory effects in horses. Of the two tested PDE inhibitors, only cilostazol has been used as a thromboprophylactic drug in humans (34), with IBMX being largely an *in vitro* or *ex vivo* experimental tool. Since we and others have shown that the weaker non-selective PDE inhibitors, theophylline and pentoxifylline, are ineffective at inhibiting platelet activation when administered to horses (see text footnote 1) or tested *ex vivo* (20, 35), cilostazol has potential as a PDE inhibitor in horses. Aside from its antiplatelet effects, cilostazol also acts as vasodilator (36). Thus, this drug may have useful rheologic effects in conditions other than EHV-1 infection, such as recurrent airway obstruction and laminitis (37, 38).

We observed that the DMSO diluent for the PDE inhibitors enhanced microvesiculation or fragmentation of equine platelets in the presence of EHV-1, but not thrombin. The absence of this effect in thrombin-stimulated platelets suggests that it is not a consequence of activation *per se*, but requires the presence of the virus. The reason for this finding is unknown, but it is possible that DMSO alters the platelet membrane, rendering it more susceptible to EHV-1 binding, which then causes microvesiculation or fragmentation, or vice versa. Because fragmentation or microvesiculation could increase the surface area available to support thrombosis, it would be prudent to avoid the use of a DMSO vehicle when administering cilostazol to horses.

Inhibition of platelet activation in horses exposed to or infected with EHV-1 may have desirable consequences in addition to preventing virus-associated thrombosis. Platelets are now known to act as an integral part of the innate immune system beyond their role in hemostasis (39). Platelets express receptors that recognize virus peptides and viruses, such as human immunodeficiency virus and Dengue virus, bind to or are internalized by platelets (25, 40). This could serve as a means for clearing virus, presenting viral antigen to T cells and enhancing antiviral immunity. Alternatively, virus endocytosis by platelets could be a provirus response, with platelets acting to protect the virus from immune destruction and as a source of infection for other cells that interact with platelets or clear them from the circulation (39, 40). Platelets also promote inflammation by secreting vasoactive and inflammatory mediators, and recruit leukocytes to endothelial cells through interactions with P-selectin on activated platelets and P-selectin glycoprotein ligand-1 on leukocytes (39). Experimental studies have shown that inhibition of P-selectin reduces inflammation in experimental murine models (13). Because we have shown that EHV-1 associates with platelets, we speculate that drugs that inhibit platelet activation and P-selectin expression may not only act as antithrombotics but also inhibit virus dissemination or virus-induced inflammatory responses.

In conclusion, our results indicate that heparin (UFH and LMWH) and the PDE3 inhibitor, cilostazol, may be useful adjunctive or prophylactic drugs to help prevent or minimize the likelihood of abortion and EHM in horses infected with or exposed to EHV-1. However, these drugs are not without side-effects and can be quite costly (LMWH and likely cilostazol). Thus, further studies are required to extend our *ex vivo* results and confirm that these drugs are effective at inhibiting EHV-1-induced platelet activation when administered to horses.

## Author Contributions

Study conception: TS; experimental design: TS, PS, and MZ; data acquisition, analysis and interpretation: TS, PS, MZ, and MB; performance and analysis of experiments: TS, PS, MZ, and MB; manuscript preparation: TS; and manuscript revision: PS, MZ, and MB.

## Conflict of Interest Statement

The authors declare that the research was conducted in the absence of any commercial or financial relationships that could be construed as a potential conflict of interest.

## Footnotes

^1^Hernandez D, Yeo WM, Brooks MB, Ness SL, Divers TJ, Stokol T. Effects of various antiplatelet drugs on ex vivo platelet activation induced by equine herpesvirus type 1. *Am J Vet Res* (2016).

## Supplementary Material

Figure S1 **Chondroitin sulfate (CS) has no effect on thrombin or EHV-1-induced platelet activation, as measured by P-selectin expression with flow cytometry**. P-selectin expression was quantified in response to thrombin (0.15 U/mL, light gray columns) or RacL11 (dark gray columns) and Ab4 (black columns) strains of EHV-1 at 1 PFU/cell as the percentage of gated platelets in the absence or presence of 5 μg/mL CS as a negative control for UFH and LMWH (*n* = 3). Columns represent medians with superimposed individual data points. The number of replicates was insufficient to perform statistical analysis.
