# Corrigendum: Unfractionated and Low-Molecular-Weight Heparin and the Phosphodiesterase Inhibitors, IBMX and Cilostazol, Block *Ex Vivo* Equid Herpesvirus Type-1-Induced Platelet Activation

**DOI:** 10.3389/fvets.2017.00010

**Published:** 2017-02-10

**Authors:** Tracy Stokol, Priscila B. S. Serpa, Muhammad N. Zahid, Marjory B. Brooks

**Affiliations:** ^1^Department of Population Medicine and Diagnostic Sciences, Cornell University, Ithaca, NY, USA

**Keywords:** equine, EHV-1, thrombin generation, thrombosis, P-selectin, flow cytometry, horse

The spelling of Priscila B. D. Serpa is wrong. The correct is **Priscila B. S. Serpa** (from full name Priscila Beatriz da Silva Serpa). The authors apologize for the mistake. This error does not change the scientific conclusions of the article in any way.

The original article has been updated.

## Conflict of Interest Statement

The authors declare that the research was conducted in the absence of any commercial or financial relationships that could be construed as a potential conflict of interest.

