# Supplementary figures and images for: Unfractionated and Low-Molecular-Weight Heparin and the Phosphodiesterase Inhibitors, IBMX and Cilostazol, Block Ex Vivo Equid Herpesvirus Type-1-Induced Platelet Activation
Source: Front Vet Sci. 2016 Nov 17;3:99. doi: 10.3389/fvets.2016.00099 (PMC5112437; doi:10.3389/fvets.2016.00099)

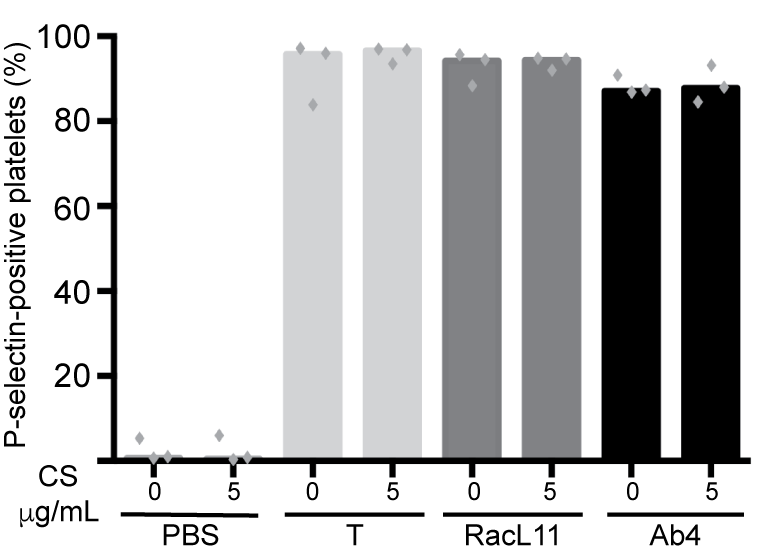

Supplement: Figure S1 — Chondroitin sulfate (CS) has no effect on thrombin or EHV-1-induced platelet activation, as measured by P-selectin expression with flow cytometry. P-selectin expression was quantified in response to thrombin (0.15 U/mL, light gray columns) or RacL11 (dark gray columns) and Ab4 (black columns) strains of EHV-1 at 1 PFU/cell as the percentage of gated platelets in the absence or presence of 5 μg/mL CS as a negative control for UFH and LMWH (n = 3). Columns represent medians with superimposed individual data points. The number of replicates was insufficient to perform statistical analysis. [file Image_1.TIF]
